# Macular vessel density before and after panretinal photocoagulation in patients with proliferative diabetic retinopathy

**DOI:** 10.1186/s40942-022-00369-1

**Published:** 2022-03-14

**Authors:** Ahmed Shawkat Abdelhalim, Mohamed Farouk Sayed Othman Abdelkader, Mohamed Salah El-Din Mahmoud, Asmaa Anwar Mohamed Mohamed

**Affiliations:** Ophthalmology Department, Faculty of Medicine, Minia University Hospital, Minia University, El-Minia, 61519 Egypt

**Keywords:** OCTA, Panretinal photocoagulation, Macular vessel density, PDR

## Abstract

**Introduction:**

Diabetic retinopathy (DR) is microangiopathy causing ischemia leading to proliferative diabetic retinopathy and macular edema. Panretinal photocoagulation (PRP) reverses the ischemia leading to regression of neovessels. Most previous studies showed the large vessel effects of PRP, while optical coherence tomography angiography (OCTA) allowed noninvasive quantification of microvascular retinal changes.

**Aim:**

To study the effect of PRP on microvascular retinal vessels in a detailed manner at different retinal and choroidal levels using OCTA.

**Patients and methods:**

This study was a prospective interventional study. 30 eyes of 18 diabetic patients with PDR were included. All patients were evaluated clinically and with OCTA (Avanti RTVue-XR system, Optovue) to evaluate superficial and deep vessels density (VDs), choroidal flow, and FAZ area before PRP (base line) and 1 month and 6 months after PRP.

**Results:**

PRP improved vessels density at superficial (SCP), deep (DCP), and choriocapillaris levels. Foveal vessel density at SCP and DCP were statistically significantly increased. SCP was 28.76 ± 2.56 at base line and was increased to 29.84 ± 2.47 and 30.89 ± 2.20 after 1 month and after 6 months, respectively. DCP was 34.08 ± 5.59 at base line and was increased to 34.93 ± 5.66 and 36.09 ± 5.62 after 1 month and after 6 months, respectively. Foveal choriocapillaris was statistically significantly increased from 63.04 ± 2.66 at base line to 63.48 ± 2.65 and 63.98 ± 2.78 after 1 month and 6 months, respectively. Choroidal flow was increased from 1.74 ± 0.07 at base line to 1.75 ± 0.09 at 1 month which was nonsignificant (P = 0.72), but it was significantly increased to 1.87 ± 0.27 6 months after PRP (P = 0.009). FAZ area was significantly improved after PRP. FAZ area was decreased from 0.56 ± 0.27 at base line to 0.50 ± 0.21 after 1 month and to 0.46 ± 0.21 after 6 months.

**Conclusion:**

OCTA parameters were significantly improved by PRP in PDR patients, possibly due to redistribution of blood in occluded capillary plexuses.

*Trials registry*: NCT04976361.

## Introduction

The global prevalence of diabetes mellitus (DM) in 2019 is estimated to be 9.3% (463 million people) [1].

Diabetic retinopathy (DR) is microangiopathy characterized by capillary non-perfusion, microaneurysms (MAs), and retinal ischemia [2]. It may cause many complications, such as diabetic macular edema (DME) and diabetic macular ischemia (DMI) [3].

Capillary ischemia decreases the nutrition of the retina and causes hypoxia which results in increased level of vascular endothelial growth factor (VEGF), which promotes angiogenic responses causing both neovascularization (proliferative diabetic retinopathy, PDR) and vascular permeability (macular edema) [4].

Panretinal photocoagulation (PRP) is the standard treatment for proliferative diabetic retinopathy (PDR). It improves oxygenation of the ischemic retina. Destruction of the highly active photoreceptor cells is the suggested mechanism of action for PRP. Subsequently, production of VEGFs, the key player in neovascularization process, is reduced leading to regression of new vessels [5].

PDR eyes have an overall lower blood flow than normal or non-PDR eyes, parallel to the higher level of retinal ischemia and disease severity. With regression of these neovascular and shunt vessels following PRP, normalization of flow in the macula may reverse the ischemia and decrease the stimulus for new blood vessel formation. Closure of intraretinal microvascular abnormalities and neovascularization would theoretically increase overall resistance to flow and, combined with the constriction of the large vessel in response to increased oxygen in the inner retina, collectively decrease the overall blood flow [6].

While most previous studies have explored the large vessel effects of PRP, the development of optical coherence tomography angiography (OCTA) allowed the study of microvascular retinal changes in a detailed manner. It is a non-invasive modality that allows vascular mapping with high speed and quality and promotes visualization of vascular system in different retinal and choroidal levels. Several studies have demonstrated the competence of OCTA in the quantification of microvascular density, choroidal flow area, and foveal avascular zone (FAZ) area in diabetic patients [7]. The aim of this work is to study the effects of PRP on the macula of eyes with high-risk PDR using OCTA.

## Patients and methods

### Study design and population

In this prospective interventional study, 30 eyes of 18 patients with type 2 DM with PDR were included. All patients were recruited, examined, treated, and followed up in the outpatient clinic of Ophthalmology Department, Minia University Hospital between March 2019, and April 2021. The study was approved by the Local Research Ethics Committee, Faculty of Medicine, Minia University Hospitals. A written informed consent was obtained from all patients and the study was adherent to the tenets of Declaration of Helsinki.

Inclusion criteria were treatment-naive PDR patients diagnosed clinically and by the presence of neovascularization on optic disc (NVDs) or elsewhere (NVEs) on fluorescein angiography. All patients had high risk PDR.

Eyes with one or more of the following criteria were excluded from the study: (1) significant media opacity decreasing image quality (corneal opacity, dense cataract, and vitreous hemorrhage); (2) significant macular edema ($\ge$ 300 µm) to overcome errors in segmentation or the need for treatment by intravitreal injection of anti-VEGF; (3) patients with glaucoma, uveitis, other maculopathies, optic neuropathy; (4) previous intraocular surgery and trauma; (4) history of previous treatment for diabetic retinopathy including intravitreal injection of anti-VEGFs, laser, or vitrectomy; (5) incomplete follow up; and (6) Scans with low signal strength index (SSI; < 50), presence of blink artifacts, and poor fixation leading to motion artifacts.

### Ophthalmic evaluation

All patients were subjected to full ophthalmological evaluation including history taking, anterior segment examination using slit-lamp biomicroscopy, intraocular pressure measurement (IOP) using slit-lamp mounted Goldman applanation tonometry, refraction, measurement of best corrected visual acuity (BCVA) using Snellen chart (converted to Log MAR), and fundus examination using a 78D Volk lens with slit lamp, and binocular indirect ophthalmoscopy, after pupillary dilatation with tropicamide 1% eye drops. Fundus photography (colored & fluorescein angiography) was performed using IMAGE net 2000 fundus camera (TOPCON Corporation, Japan).

### Optical coherence tomography angiography image acquisition (OCTA)

The instrument used for OCTA image acquisition is a spectral domain OCT system (Avanti RTVue-XR; Optovue, Fremont, CA), which uses the AngioVue Imaging System.

It is a dual-modality OCT-based system, imaging both structure and ocular microvasculature. Split-spectrum amplitude-decorrelation angiography (SSADA) with optical software (Angio Vue version 2017.1.0.155; Optovue, Inc.) was used to extract the OCT angiography information. Motion correction technology to minimize motion artifacts was used. The images were captured with the standard macula protocol with a scan area 6 mm × 6 mm.

AngioVue uses a simplified set of reference to make automatic segmentation to examine different vascular plexus:

- Superficial retinal capillary plexus (SCP): extended between the internal limiting membrane (ILM) and 10 μm above the inner plexiform layer (IPL).
- Deep retinal capillary plexus (DCP): extended 10 μm above the inner plexiform layer (IPL) and 10 μm below the outer plexiform layer (OPL)
- Choriocapillaris: extended from 30 to 60 μm below the retinal pigment epithelium (RPE) reference.

Microvascular changes can be quantified as vessel area density which is the retinal area occupied by blood vessels relative to total area expressed as vessels density % and can be calculated automatically from vessel density map. Foveal VD is calculated within the central 1 mm circle, and parafoveal VD is calculated in the area extending from 0.5 to 1.5 mm, while whole image VD is measured from the scan area of 6 mm × 6 mm. The FAZ area is automatically measured in mm [2] by clicking the center of the FAZ while using the nonflow function tool.

OCTA examination was performed before PRP, and after PRP by 1 month and 6 months.

### Panretinal photocoagulation (PRP)

PRP was done using visulas green laser machine 532 nm (Carl Zeiss Meditec AG Geoschwitzer Str. 51-52,07745 Jena, Germany) and Ocular mainster PRP 165 laser lens (Ocular Instruments, Bellevue, WA 98004, USA) according to Early Treatment Diabetic Retinopathy Study group study. PRP was performed in 2 sessions with an interval of 1 week in-between. Laser burns (400 microns) were spaced one laser spot size apart. Treatment was carried out posteriorly just outside the arcades and as far as possible peripherally. Laser settings were 200-micron spot size (leading to retinal laser burns of approximately 400 microns), pulse duration of 100 ms, and power of 200–250 milliwatts. The goal was to produce burns that were grey in color (not dense white burns) with a total number of 2000 burns. Follow up visits were at 1 and 6 months after base line measures.

### Statistical analysis

Statistical analysis was performed on IBM SPSS version 25. Qualitative variables were presented as frequency and percentage, and quantitative variables were presented as mean and standard deviation (SD). Univariate repeated measures analysis of variance (ANOVA) was used to examine the changes in all the record parameters comparing the three visits. Post-hoc Tukey LSD analyses were run for pairwise comparison. P-value < 0.05 was considered to be statistically significant.

## Results

### Demographic data

This study included 30 eyes with high-risk PDR of 18 patients with type 2 DM (12 patients had bilateral PDR and 6 patients had unilateral PDR). 10 patients were males, and 8 patients were females. Their mean age was 59.27 ± 4.2 years (range: 52–65 years). Mean duration of DM was 17.3 + 3.01 years. All eyes were phakic. Controlled hypertension was present in 7 patients. HbA1c ranged from 6.8 to 9.4% with a mean of 7.93 ± 0.81.

### OCTA findings (Table 1, and Figs. 1, 2)

**Table 1:** Changes in VD and choroidal flow at baseline, 1 month, and 6 months follow up

	Repeated measures ANOVA Baseline	1 month	6 months	p-value	Pairwise Baseline Vs 1 month	Comparison Baseline Vs 6 months
	Mean ± SD	Mean ± SD	Mean ± SD			
SCP whole image	44.41 ± 2.64	45.45 ± 2.62	46.37 ± 2.58	0.000	0.000	0.000
SCP fovea	28.76 ± 2.56	29.84 ± 2.47	30.89 ± 2.20	0.000	0.000	0.000
SCP parafovea	43.92 ± 3.87	44.48 ± 3.96	45.58 ± 4.01	0.000	0.000	0.000
DCP whole image	48.74 ± 2.31	49.64 ± 2.28	50.71 ± 2.17	0.000	0.000	0.000
DCP fovea	34.08 ± 5.59	34.93 ± 5.66	36.09 ± 5.62	0.000	0.000	0.000
DCP parafovea	51.98 ± 1.99	52.87 ± 2.04	54.07 ± 2.02	0.000	0.000	0.000
Choriocapillaris VD Whole image	63.24 ± 1.71	63.08 ± 3.62	64.01 ± 1.70	0.166	0.789	0.0001
Choriocapillaris VD fovea	63.04 ± 2.66	63.48 ± 2.65	63.98 ± 2.78	0.0001	0.0001	0.0001
Choriocapillaris VD parafovea	63.18 ± 1.80	63.51 ± 1.86	63.89 ± 1.87	0.0001	0.0001	0.0001
Choroidal flow	1.74 ± 0.07	1.75 ± 0.09	1.87 ± 0.27	0.008	0.722	0.009
Retinal thickness fovea	275.90 ± 55.64	283.67 ± 55.80	277.83 ± 59.61	0.006	0.000	0.478
Retinal thickness parafovea	343.33 ± 53.48	332.03 ± 43.54	338.30 ± 50.56	0.0.244	0.105	0.332

**Fig. 1:**
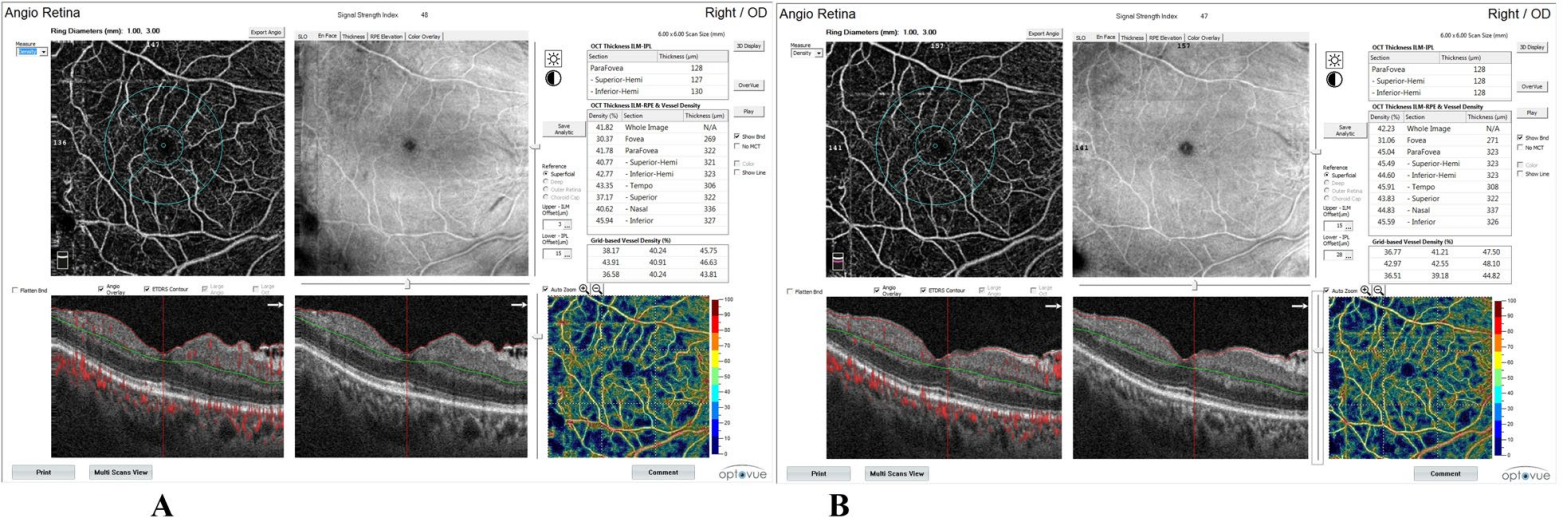
Rt eye OCTA scan (6 ×  6 mm^2^) of a patient with PDR at the level of the superficial retinal plexus centered on the macula. Vessel density is presented on the OCT thickness ILM-RPE, vessel density map, and on grid-based vessel density (%) (upper right) with color-coded flow density map (lower right), where areas with warm colors have greater flow (**A** at base line, **B** at 6 months following PRP)

**Fig. 2:**
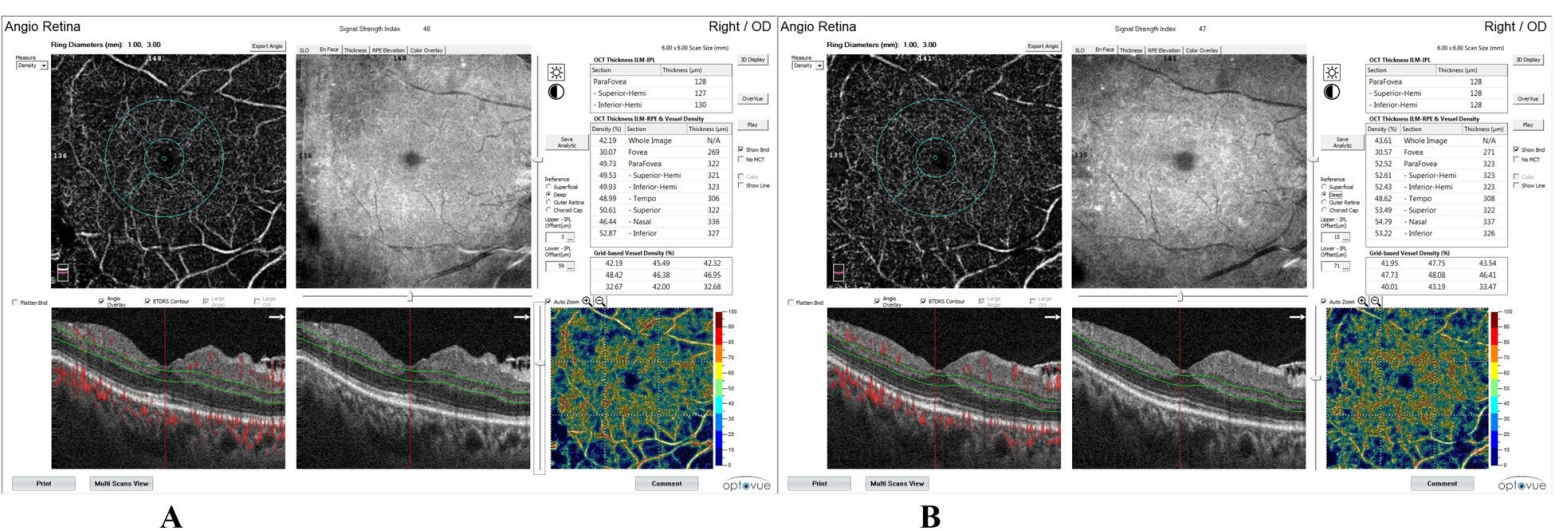
Rt eye OCTA scan (6 ×  6 mm^2^) of a patient with PDR at the level of the deep retinal plexus centered on the macula. Vessel density is presented on the OCT thickness ILM-RPE, vessel density map, and on grid-based vessel density (%) (upper right) with color-coded flow density map (lower right), where areas with warm colors have greater flow (**A** at base line, **B** at 6 months following PRP)

Foveal vessel density (VD) at SCP and DCP was statistically significantly increased. SCP was 28.76 ± 2.56 at base line and was increased to 29.84 ± 2.47 and 30.89 ± 2.20 after 1 month and after 6 months, respectively. DCP was 34.08 ± 5.59 at base line and was increased to 34.93 ± 5.66 and 36.09 ± 5.62 after 1 month and after 6 months, respectively. Parafoveal vessel density at SCP and DCP were statistically significantly increased as SCP at base line was 43.92 ± 3.87, after 1 month 44.48 ± 3.96, and after 6 months 45.58 ± 4.01. Parafoveal DCP at base line was 51.98 ± 1.99, after 1 month 52.87 ± 2.04, and after 6 months 54.07 ± 2.02. Vessel density in the whole image at SCP and DCP was statistically significantly increased. Whole image SCP was 44.41 ± 2.64 at base line and was increased to 45.45 ± 2.62 and 46.37 ± 2.58 after 1 month and after 6 months, respectively. Whole image DCP was 48.74 ± 2.31 at base line and was increased to 49.64 ± 2.28 and 50.71 ± 2.17 after 1 month and after 6 months, respectively.

Foveal choriocapillaris were statistically significantly increased as choriocapillaris VD was 63.04 ± 2.66 at base line and was increased to 63.48 ± 2.65 and 63.98 ± 2.78 after 1 month and 6 months, respectively. Parafoveal VD of choriocapillaris was statistically significantly increased as choriocapillaris VD at base line was 63.18 ± 1.80, after 1 month 63.51 ± 1.86, and after 6 months 63.89 ± 1.87. Choriocapillaris VD in the whole image was statistically significantly increased from 63.24 ± 1.71 at base line to 64.01 ± 1.70 after 6 months. Choriocapillaris VD in the whole image at 1 month was not statistically different from that of base line.

Choroidal flow area was increased from 1.74 ± 0.07 at base line to 1.75 ± 0.09 at 1 month which was nonsignificant (P = 0.72), but it was significantly increased to 1.87 ± 0.27 at 6 months after PRP (P = 0.009).

Retinal thickness at the fovea was significantly increased from 275.90 ± 55.64 at base line to 283.67 ± 55.80 at 1 month (P = 0.00), and then decreased to 277.83 ± 59.61 6 months after PRP. Comparing foveal retinal thickness between base line and 6 months, it was found nonsignificant (P = 0.47). Parafoveal retinal thickness changed from 343.33 ± 53.48 at base line to 332.03 ± 43.54 after 1 month and to 338.30 ± 50.56 after 6 months which were statistically nonsignificant.

As regards FAZ area and visual acuity, there were significant improvement after PRP.

**Fig. 3:**
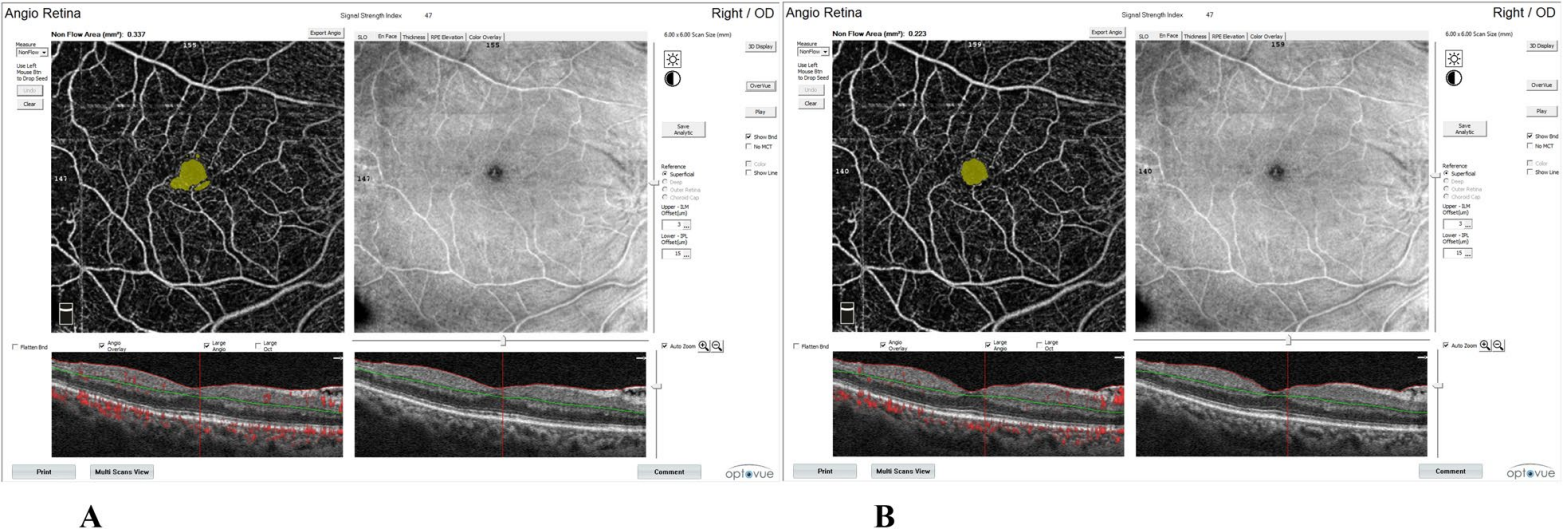
OCTA of FAZ area showing constriction following PRP (**A** at baseline and **B** 6 months following PRP)

FAZ area was decreased significantly from 0.56 ± 0.27 at base line, to 0.50 ± 0.21 after 1 month and to 0.46 ± 0.21 after 6 months. BCVA was increased significantly from 0.88 ± 0.10 at base line to 0.71 ± 0.19 after 1 month, and to 0.51 ± 0.12 after 6 months (Table 2, Fig. 3).

**Table 2:** Changes in FAZ area and visual acuity (log MAR) at base line, 1 month, and 6 months follow up

	Baseline	1 Month	6 Months	p-value	Base line VS 1 month	Base line VS 6 months
FAZ area	0.56 ± 0.27	0.50 ± 0.21	0.46 ± 0.21	0.000	0.006	0.000
Visual acuity	0.88 ± 0.10	0.71 ± 0.19	0.51 ± 0.12	0.000	0.000	0.000

## Discussion

Diabetic retinopathy is the most common microvascular complication of DM. In its natural history, capillary nonperfusion and retinal ischemia cause progression from the nonproliferative form of the disease to PDR, which is one of the main causes of vision loss in diabetic patients [8].

PRP in cases of PDR is known to cause the reduction of cytokines, such as VEGF, interleukin-6, intercellular adhesion molecule-1, and monocyte chemotactic protein-1 which stimulate vascular permeability and capillary dropout and reduce the perfusion of the posterior pole increasing the macular ischemia and enlarging FAZ area [9].

In PDR, PRP is believed to improve inner retinal oxygen delivery with consequent decreased angiogenic drive and regression of neovascularization. OCTA allows the study of how these vessel hemodynamic changes affected blood flow distribution in the macular microvasculature and its sublayers [10].

Many studies investigated the effect of PRP on retinal capillary circulation and perfusion. The most shortcoming of them is the utilization of fluorescein angiography to quantify retinal ischemia which has a limited capability to evaluate the deep retinal capillary plexus due to the superimposition of the superficial capillary plexus over the deep capillary plexus as well as masking by leakages and hemorrhages [11].

OCTA is a non-invasive depth-resolving technique allowing retinal vasculature mapping at different capillary plexuses levels [12]. Few studies have investigated the laser effects on OCTA microvascular parameters as microvascular density, capillary non-perfusion, FAZ and choroidal flow in diabetic patients.

In this study, we used OCTA to study the microvascular changes following PRP in patients with high-risk PDR and found statistically significant improvement in vessels density in SCP and DCP for fovea, para-fovea, and whole image at 1 month and 6 months follow up periods. This may be attributed to reorganization of capillary networks following PRP [13].

Our results disagreed with Lorusso et al., 2019 who conducted a clinical study on 18 eyes of 14 diabetic patients with measurement of BCVA, FA, OCT, and OCTA at baseline. Patients were treated by PRP using frequency-doubled Nd: YAG pattern scan laser and followed up at 1 month and 6 months. They concluded that OCTA parameters were not significantly affected by laser treatment at 1-month and 6-months follow-up. In the study of Lorusso et al., 2019, the age of patients was less than our study, the number of eyes was less than our study, and they included patients with type 1 DM and type 2 DM. Studies showed that the short-pulse laser delivery system results in less-destructive and less anti-ischemic effects at the molecular level [14, 15].

Faghihi et al., 2020 used OCTA to study macular vascular density, FAZ, and choroidal flow following a conventional PRP treatment in 39 eyes of patients with very severe non-proliferative and early proliferative PDR [16]. In Faghihi et al., 2020 study, vascular density in the foveal and parafoveal region did not change statistically significantly although a trend to increasing vascular density in the foveal region was shown, both in SCP and DCP. However, these trends were different in the parafoveal area; a 1-month post PRP decrease in vascular density was documented in the parafoveal area in the SCP and DCP; in the 6 months follow up after PRP, statistically non-significant increases were recorded in SCP and DCP in the parafoveal area. In our study, we treated patients with high-risk PDR and reported a statistically significant improvement in vessels density in 1 month and 6 months follow up periods, in SCP and DCP for fovea and para-fovea.

Fawzi et al., 2019 used OCTA to study 10 eyes of 10 subjects with high-risk PDR immediately before, 1 month and 3–6 months following PRP, using 3 × 3 mm OCTA scan at each visit. Fawzi et al., 2019 didn’t find a significant change in vascular density parameters following PRP, although they suggested an overall redistribution of blood flow to the posterior pole following PRP [13]. In addition to studying vessel density and choroidal flow, Fawzi et al. 2019 studied electrical circuit model of retinal circulation which studied the hemodynamics of the retinal vasculature network in a mathematical way which showed that PRP would have associated with increased macular flow which was confirmed in our study.

Both Fawzi et al. 2019 and Faghihi et al., 2020 used the 3 × 3 mm scan while in our study, a wider 6 × 6 mm angiography macular scan was taken which included the entire posterior pole.

Regarding thickness of fovea, it was increased after 1 month and was decreased again after 6 months of follow up. Many studies reported the occurrence of macular edema after PRP which resolved later. Blood-retinal barrier disruption might be the cause of this macular edema [17, 18]. On other hand, non-significant changes were found regarding thickness of parafoveal area in our study.

The present study found a statistically significant difference in choriocapillaris VD and choroidal flow between baseline and follow up periods. Takahashi et al. using laser Doppler flowmetry, reported a significant rise in subfoveal choroidal flow 1 month after PRP in cases of non-clinically significant macular edema. They postulated 2 mechanisms for the increased choroidal flow. One mechnism was the redistribution of choroidal blood flow from the obliterated peripheral capillaries to the posterior pole, and the other was choroidal inflammation induced by PRP [19, 20].

Zhao et al., 2021 evaluated choroidal and retinal microvasculature with OCTA after PRP for patients with PDR or severe non-proliferative diabetic retinopathy and concluded that OCTA demonstrated redistribution of choroidal circulation from the periphery to the macula after PRP [21].

Our study illustrated significant improvement of FAZ area after PRP which agreed with Faghihi et al., 2020 who showed that the FAZ area was constricted and became significantly more circular 6 months following PRP [22]. It has been shown that deterioration of diabetic retinopathy may be correlated with more irregular FAZ due to capillary occlusion or altered blood flow [23].

Previous studies using OCTA suggested that FAZ enlargement, especially in the deep capillary plexus, was common among patients with DM, regardless of concomitant diabetic retinopathy [24, 25] FAZ enlargement in diabetic retinopathy has been confirmed through OCTA and FA [26]. Changes of FAZ area could be an important marker for macular status monitoring before or after treatment in diabetic patients [10]. Diabetic microangiopathy with consequent capillary occlusion might be the primary cause for FAZ enlargement in patients with DM [27]. Improved and more effective flow in the remaining capillaries of the macula could be a direct result of closure of the peripheral neovascular and intraretinal microvascular abnormalities, with more effective perfusion of the posterior pole.

Further studies with a larger number of patients and longer follow up periods are needed to confirm our results.

In conclusion, in this study, we found that OCTA parameters were significantly affected by PRP in patients with high-risk PDR, possibly due to redistribution and reperfusion of occluded capillary plexuses.

## Data Availability

Data is available upon request from author.
